# Functional brain connectivity correlates of pain relief during virtual reality exposure in cancer patients

**DOI:** 10.3389/fpain.2026.1730496

**Published:** 2026-07-15

**Authors:** Somayeh B. Shafiei, Saeed Shadpour, Oscar de Leon-Casasola

**Affiliations:** 1 The Intelligent Cancer Care Laboratory, Department of Urology, Roswell Park Comprehensive Cancer Center, Buffalo, NY, United States; 2 Department of Animal Biosciences, University of Guelph, Guelph, ON, Canada; 3 Division of Pain Medicine, Department of Anesthesiology, Roswell Park Comprehensive Cancer Center, Buffalo, NY, United States

**Keywords:** cancer pain, virtual reality, functional near-infrared spectroscopy (fNIRS), functional brain connectivity, non-pharmacological pain management

## Abstract

**Introduction:**

Virtual reality (VR) has shown promise for pain relief, including in cancer-related contexts, yet its underlying neurophysiological correlates remain unclear. Understanding brain-based correlates is important for evaluating VR programs and informing personalized pain management. Functional connectivity reflects coordinated activity among pain-related brain regions.

**Methods:**

This study examined associations between changes in functional connectivity and pain reduction during VR exposure using functional near-infrared spectroscopy (fNIRS) in cancer patients with chronic neuropathic and/or somatic pain. In an IRB-approved study, 41 cancer patients underwent a VR distraction session using the Oceania application on a Meta Quest® headset. Continuous fNIRS signals focused on oxygenated hemoglobin (HbO) were recorded. Pain intensity was assessed before and after VR using the FACES Pain Scale–Revised. Functional connectivity was estimated using coherence analysis, and associations with pain reduction were evaluated using Pearson correlation with false discovery rate correction.

**Results:**

Extracted functional connectivity features showed significant negative correlations with pain reduction, particularly between channels spanning prefrontal cortex (PFC), bilateral parietal lobes [including the primary somatosensory cortex (S1)], and superior frontal gyrus (SFG). Strongest associations were observed between PFC and SFG (r = –0.43, *p* = 0.005), and within SFG (r = –0.42, *p* = 0.006). HbO activity at the PFC was significantly associated with pain reduction (r = –0.32, *p* = 0.044).

**Discussion:**

Decreased functional connectivity in specific cortical regions was observed in association with short-term pain reduction during VR exposure. Findings suggest that functional connectivity features may represent candidate neurophysiological correlates of pain changes in immersive VR contexts.

## Introduction

Cancer-related pain is prevalent and debilitating, greatly reducing quality of life (1, 2). Virtual reality (VR) is emerging as a non-pharmacologic pain relief approach through attention redirection and immersive distraction (3–5). VR may relieve pain by creating an immersive environment that redirects attention away from nociceptive input and pain perception, while promoting relaxation and emotional engagement. In oncology populations, VR has also been explored as a supportive intervention for cancer-related symptom management, including in advanced cancer patients receiving home-based care (6). Although VR's analgesic effects are behaviorally documented, the underlying brain mechanisms, especially in cancer patients, remain less understood (4). Functional near-infrared spectroscopy (fNIRS) provides a non-invasive means to assess brain activation and functional connectivity during pain modulation, offering mechanistic insights to personalize VR therapies (4).

Pain perception involves distributed brain regions that process sensory, cognitive, and emotional components of pain (7, 8). Somatosensory and parietal regions contribute to sensory localization and intensity processing, while the prefrontal cortex and superior frontal regions are involved in attention, cognitive control, and top-down modulation of pain (7, 9). Deeper regions such as the anterior cingulate cortex and insula are also important components of salience and affective pain-processing networks, although they are not directly accessible with fNIRS (10, 11). By redirecting attention and promoting immersion, VR may alter activity and connectivity within these cortical pain-modulatory networks (12, 13).

fNIRS is a non-invasive neuroimaging technique that monitors cortical hemodynamics during real-world tasks. By measuring oxygenated hemoglobin (HbO) changes, it reveals how specific brain regions are activated and interact during pain perception and modulation.

Coherence analysis measures frequency-specific coupling of fNIRS signals (e.g., HbO) between brain regions, providing an estimate of functional connectivity, or coordinated activity among regions within a brain network (14, 15).

In pain, altered functional connectivity may indicate disrupted or compensatory brain responses, whereas a shift toward pain-free patterns may signal effective relief (8, 16, 17). Recognizing these patterns helps identify neurophysiological biomarkers of treatment response and informs the development of personalized, brain-based pain management strategies, especially for chronic or treatment-resistant cases.

Altered functional connectivity is common in chronic pain, reflecting disrupted interactions between pain-processing and modulatory brain regions (8, 18, 19). In cancer care, multimodal pharmacologic pain management is often limited by side effects, tolerance, incomplete response, or patient preference, highlighting the need for effective non-invasive complementary approaches (20).

Understanding the neural correlates associated with pain changes during immersive VR exposure may help clarify how VR-based interventions influence pain-related brain networks and may inform future development of personalized digital symptom-management strategies.

Therefore, this exploratory single-arm study examined whether short-term pain reduction during a single VR-based relaxation/distraction session is associated with fNIRS-derived cortical activation and functional connectivity changes in cancer patients with chronic pain. We focused on oxygenated hemoglobin activity and coherence-based functional connectivity across cortical regions involved in pain perception, attention, and cognitive modulation.

## Methods

Forty-one adult cancer patients (33 males, 8 females; mean age = 62 ± 11.3 years; BMI = 29 ± 8.2) with chronic cancer-related pain were recruited from the outpatient clinic of the Division of Pain Medicine at Roswell Park Comprehensive Cancer Center. All patients were receiving multimodal pharmacological therapy but reported inadequate pain relief. Cancer type was defined based on the combination of primary disease site and histologic diagnosis and is summarized in Table 1. Participants experienced chronic cancer-related pain lasting longer than 9 months at the time of enrollment, with neuropathic and/or somatic components, consistent with mixed-mechanism cancer pain commonly observed in oncology populations. Although these pain states originate from peripheral mechanisms, chronic neuropathic and somatic pain are increasingly recognized to involve altered large-scale brain network dynamics, often referred to as *brain network pain*.

**Table 1:** Primary cancer site and histologic diagnosis of study participants*.

Characteristic	n (%)
Primary disease site	
Lung	10 (24.4)
Prostate	4 (9.8)
Kidney	4 (9.8)
Bone marrow	4 (9.8)
Breast	3 (7.3)
Bone	2 (4.9)
Tonsil	2 (4.9)
Cervical lymph nodes	1 (2.4)
Chest lymph nodes	1 (2.4)
Thoracic vertebrae	1 (2.4)
Stomach	1 (2.4)
Small intestine	1 (2.4)
Esophagus	1 (2.4)
Bladder	1 (2.4)
Oropharynx	1 (2.4)
Pancreas	1 (2.4)
Submandibular salivary gland	1 (2.4)
Shoulder	1 (2.4)
Endometrium	1 (2.4)
**Histologic diagnosis**	
Adenocarcinoma, NOS	19 (46.3)
Multiple myeloma	4 (9.8)
Renal cell carcinoma/renal cell carcinoma, sarcomatoid	4 (9.8)
Squamous cell carcinoma/squamous cell carcinoma, NOS	4 (9.8)
Diffuse large B-cell lymphoma	2 (4.9)
Adenocarcinoma	1 (2.4)
Chronic lymphocytic leukemia	1 (2.4)
Acute lymphocytic leukemia	1 (2.4)
Acute lymphoblastic leukemia	1 (2.4)
Neuroendocrine carcinoma	1 (2.4)
Transitional cell carcinoma	1 (2.4)
Adenoid cystic carcinoma	1 (2.4)
Malignant peripheral nerve sheath tumor	1 (2.4)

*Percentages were calculated using the full sample size (*N* = 41) and may not total exactly 100% because of rounding. Cancer type was defined based on primary disease site and histologic diagnosis. Cancer stage and detailed oncologic treatment status were not consistently available and are therefore not reported.

Participants had no neurological/psychiatric disorders, brain metastases, head implants, or pregnancy, and removed items obstructing fNIRS contact. Those with hypersensitivity to flashing lights/motion, impaired stereoscopic vision, severe hearing loss, or nausea/dizziness risk were excluded.

Participants completed a 9-minute immersive VR session [Oceania program, Meta Store (21)] while fNIRS data were recorded using the Dual Brite system with Oxysoft 3.3 software (Artinis Medical Systems®, Netherlands). The VR intervention consisted of a single, immersive relaxation/distraction session delivered through the Oceania application using a Meta Quest® headset. The program presented a calming ocean-based virtual environment designed to promote relaxation and attentional engagement away from pain. Participants wore the VR headset while seated and were instructed to view and engage with the virtual environment for the full 9-minute session. No sham VR or non-VR control condition was used in this exploratory single-arm study; therefore, the analysis focused on within-subject changes in pain ratings and fNIRS-derived brain activation/connectivity during VR exposure relative to the pre-VR resting baseline.

**Figure 1:**
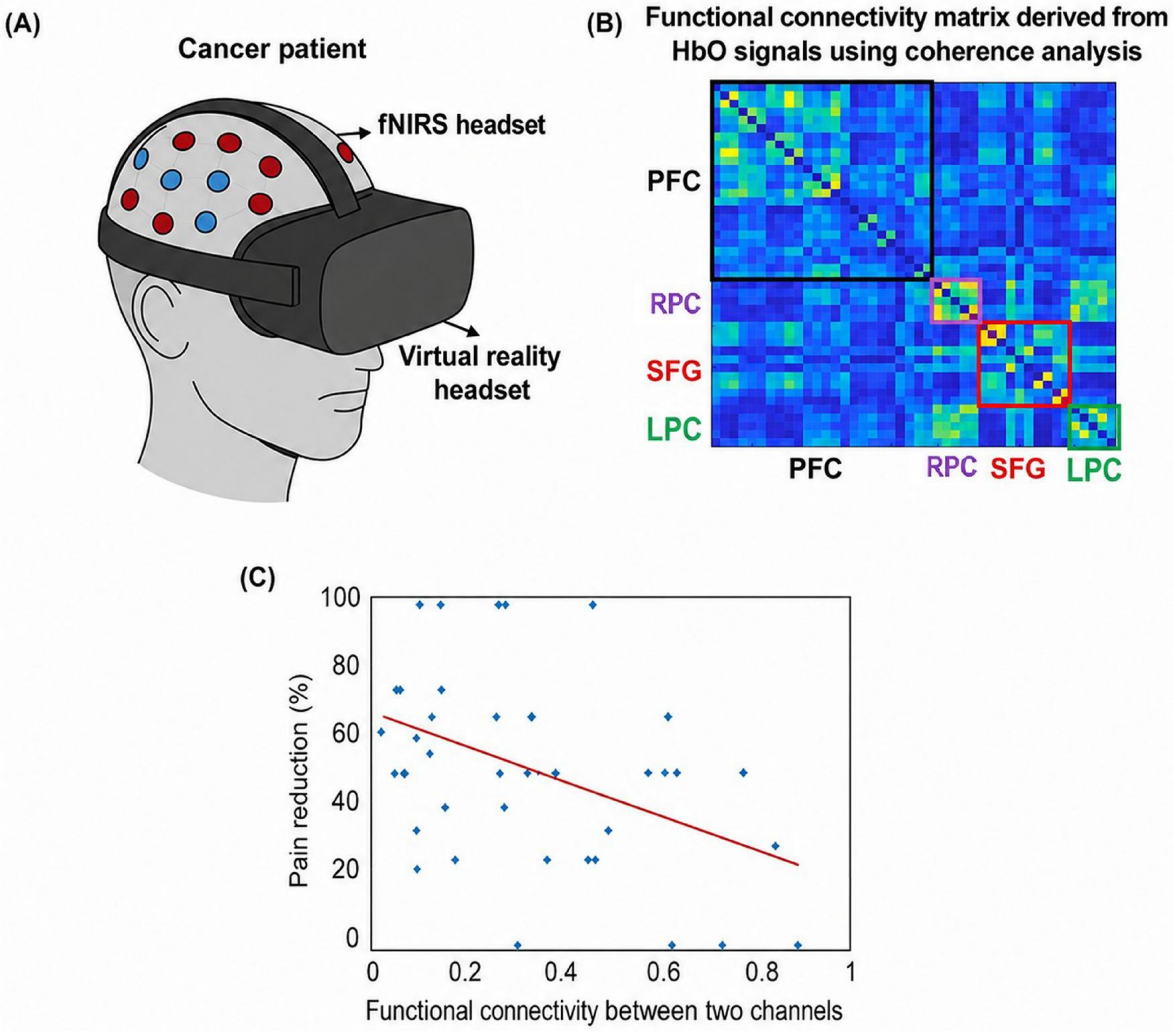
Integration of fNIRS and virtual reality, and associated brain functional connectivity findings in cancer patients with chronic pain. **(A)** Cancer patients wore a wireless fNIRS headset while engaging in the *Oceania* VR relaxation program. This element was generated using the ChatGPT 4o AI, which assisted in the creation of visual elements. **(B)** Oxygenated hemoglobin (HbO) signals were used to compute functional connectivity between and within brain regions. **(C)** Correlations between functional connectivity metrics and pain reduction were calculated.

The 44-channel setup covered multiple cortical regions (Figure 1). The parietal lobe includes superficial primary somatosensory cortex (S1) areas accessible to fNIRS, which detects hemodynamic changes up to ∼2 cm beneath the scalp, enabling monitoring of surface-level activity in pain-related regions such as S1.

Channels 1–24 were assigned to the PFC, 25–29 to the right parietal cortex (RPC), 30–39 to the SFG, and 40–44 to the left parietal cortex (LPC) (4). A 10-minute resting-state fNIRS recording was obtained prior to the VR intervention to establish baseline cortical activity.

### fNIRS signal processing

Signals were motion-corrected, bandpass-filtered (0.01–0.2 Hz), converted to HbO via the modified Beer–Lambert law (22), normalized, and baseline-corrected relative to the pre-VR resting state to isolate changes observed during VR exposure. MATLAB R2022a and the NIRS Toolbox pipeline were used for signal processing. For each subject, the pre-VR baseline mean was subtracted from VR data to quantify relative HbO changes from the intervention.

### fNIRS-based features

Average HbO amplitudes served as the activation metric, and preprocessed HbO signals were used to compute functional connectivity via coherence analysis. Magnitude-squared coherence (MSC) was computed pairwise between fNIRS channels for the HbO signals to assess functional connectivity changes during VR.

### Correlation analysis

Pain severity ratings were collected before and after VR using the FACES Pain Scale-Revised (FPS-R) tool (23), and percent reduction was calculated as $\left[\frac{\;pain_{\;pre-VR}-pain_{\;post-VR}}{\;pain_{\;pre-VR}}\right]\times 100$.

Pearson correlations were calculated between extracted metrics (mean activation and functional connectivity) and percent pain reduction, with false discovery rate (FDR) correction for multiple comparisons. Statistical significance was set at *p* < 0.05.

All channel-level and channel-pair analyses were performed across the full set of recorded fNIRS channels in an unbiased manner, and only results surviving FDR correction were reported.

### Behavioral statistical analysis

Because pain scores were not normally distributed (Shapiro–Wilk test), changes in perceived pain severity before and after the VR intervention were assessed using the Wilcoxon signed-rank test. Statistical significance was set at *p* < 0.05.

This study was designed as an exploratory investigation to examine associations between short-term pain reduction during VR exposure and fNIRS-derived cortical activation and functional connectivity in a cancer population. As such, a formal *a priori* sample size calculation was not performed. The sample size was determined pragmatically and aligns with prior fNIRS studies of pain and analgesia, which typically use moderate cohorts to explore neural mechanisms rather than confirm definitive effects.

For example, prior fNIRS studies examining pain and cortical activity during VR exposure have typically included sample sizes ranging from approximately 15 to 60 participants (24–26). Accordingly, the present findings are intended to be exploratory and hypothesis-generating, rather than confirmatory.

## Results

Average pain scores for 41 cancer patients with chronic pain were 4 ± 2.01 before VR and 2 ± 1.59 after VR. Perceived pain severity significantly decreased following the VR intervention (Wilcoxon signed-rank test, *p* < 0.001). More than 75% of participants experienced pain relief exceeding 30%, a threshold commonly considered clinically meaningful (27).

Significant negative correlations were observed between functional connectivity metrics, derived from HbO signals during VR engagement, and pain relief (Table 2), suggesting that lower functional connectivity, that is, reduced synchronization across cortical regions, is associated with greater analgesia.

**Table 2:** Significant correlations between functional connectivity during VR engagement and pain relief .

Channel Pair	Corresponding Brain Region(s)	r-value	FDR-adjusted *p*-value
1–34	PFC–SFG	−0.33	0.032
1–38	PFC–SFG	−0.42	0.006
2–38	PFC–SFG	−0.38	0.014
3–38	PFC–SFG	−0.37	0.016
15–38	PFC–SFG	−0.43	0.005
17–19	PFC–PFC	−0.31	0.049
17–23	PFC–PFC	−0.38	0.015
17–35	PFC–SFG	−0.34	0.031
22–38	PFC–SFG	−0.31	0.046
23–35	PFC–SFG	−0.35	0.023
23–43	PFC–LPC	−0.33	0.038
23–44	PFC–LPC	−0.32	0.040
25–38	RPC–SFG	−0.31	0.047
34–38	SFG–SFG	−0.42	0.006

Significant associations were observed between average HbO activation and pain reduction at two individual channels. Specifically, higher pain relief was significantly correlated with lower average HbO activation levels at channel 11 (PFC; r = –0.316, *p* = 0.044) and channel 17 (PFC; r = –0.354, *p* = 0.023). These channels were reported because they showed statistically significant correlations after FDR correction in an unbiased analysis across all recorded channels; no channels were selected *a priori*, supporting the robustness of the observed associations.

The observed correlations were of moderate magnitude (|r| ≈ 0.3–0.4), suggesting meaningful associations despite the exploratory nature of the study.

## Discussion

The primary contribution of this study is methodological, demonstrating the feasibility of integrating immersive VR with fNIRS-based functional connectivity analysis in a cancer population, rather than establishing VR-specific analgesic mechanisms. This study observed that short-term pain reduction during VR exposure in cancer patients was associated with decreased functional connectivity between the prefrontal cortex, superior frontal gyrus, and parietal lobes, as well as reduced prefrontal cortical activation. These findings suggest that immersive VR may be accompanied by changes in attentional and cortical network dynamics related to pain perception and cognitive control, although causal mechanisms cannot be inferred.

The cancer pain experienced by participants in this study comprised neuropathic and/or somatic components, reflecting mixed-mechanism chronic pain (28, 29). While these pain types originate from peripheral tissue or nerve pathology, chronic pain states are known to involve maladaptive reorganization of large-scale brain networks, including the salience network and default mode network (8, 16, 18, 30, 31). Excessive salience attribution to nociceptive input and persistent engagement of self-referential processing have been implicated in the maintenance of chronic pain, even when peripheral drivers remain stable (8, 16, 32). The reduced functional connectivity and prefrontal activation observed during VR engagement in this study are consistent with a transient suppression of these centrally mediated network processes (7, 13, 32).

The findings suggest that short-term pain reduction observed during VR exposure was associated with reduced cortical synchrony in specific brain regions. These neural observations occurred alongside a statistically significant decrease in self-reported pain severity following VR, indicating that the functional connectivity changes were observed in the context of concurrent behavioral pain changes. Accordingly, these findings should be interpreted as correlational rather than VR-specific causal effects, as the single-arm design does not allow exclusion of nonspecific factors such as expectancy, time effects, or spontaneous pain fluctuations. Chronic pain often involves increased or less flexible connectivity among pain-processing and modulatory regions (8, 32), and VR distraction may disrupt these patterns, fostering a more flexible brain network state. Reduced connectivity, particularly between prefrontal, parietal, and superior frontal regions, likely reflects a cognitive shift from internally focused pain monitoring to externally focused engagement. This aligns with evidence that decreased coupling in these networks is associated with reduced pain awareness and better analgesia (7, 9, 33). In this context, decreased synchrony may indicate effective disengagement of the pain network, consistent with reduced pain ratings. The strongest effects were seen in channels spanning the prefrontal cortex and superior frontal gyrus, with additional significant associations involving parietal regions, supporting the involvement of cortical networks related to attention, cognitive control, and pain modulation (33, 34).

Beyond changes in individual brain regions, the observed pattern of connectivity alterations aligns with known large-scale brain networks involved in chronic pain (8, 18, 35).

Although fNIRS does not directly capture activity in deep cortical structures such as the anterior cingulate cortex or anterior insula, key nodes of the salience network, prefrontal and superior frontal regions measured here are strongly functionally connected to these structures and play a central role in cognitive control, attention, and pain modulation (7, 10, 11, 33).

Significant negative correlations between pain reduction and HbO levels in two prefrontal cortex channels indicated that greater analgesia was linked to lower PFC activation, possibly reflecting reduced cognitive and emotional burden during VR. The PFC is central to pain processing, emotion regulation, and attention (7, 36), and in chronic pain, increased activity is often tied to heightened pain awareness, persistent negative thinking, and distress (8, 32). Thus, decreased PFC activation during VR may signal reduced cognitive-emotional engagement with pain, enabling deeper immersion in distraction and greater pain relief.

Prior neuroimaging studies demonstrate that VR-based analgesia is associated with reduced salience network activation and altered coupling between the default mode network and medial frontal cortex, supporting a network-level mechanism of pain relief (8, 12, 13, 37–40). This interpretation is consistent with prior neuroimaging studies using other techniques, including fMRI studies of VR analgesia showing reduced pain-related brain activity, and studies of interventional analgesia in cancer pain showing decreased salience-network connectivity after pain relief (13, 41).

Decreased inter-channel functional connectivity, particularly between the PFC and SFG, was significantly correlated with greater pain relief (e.g., channels 15–38: r = –0.429, *p* = 0.005). These regions are involved in cognitive control, attention regulation, and top-down pain modulation. Reduced connectivity may indicate temporary disengagement of control networks, enabling greater immersion in VR and less cognitive focus on pain. This shift away from self-monitoring and pain-related processing may facilitate analgesia through attentional redirection and emotional detachment.

Combined reductions in PFC activation and its connectivity with other regions indicate diminished top-down modulation during effective VR distraction. These findings support that VR influences both activation and connectivity in pain-modulatory regions, particularly the PFC (12, 42).

Reduced functional connectivity may indicate neural decoupling that enables attentional disengagement from pain, consistent with theories that diminished connectivity within cognitive and salience networks facilitate relief by limiting integration of nociceptive inputs. VR shows promise as an immersive, non-pharmacologic analgesic in cancer care, and fNIRS-derived connectivity metrics could help identify likely responders and serve as biomarkers of treatment responsiveness.

Prior research supports this interpretation, showing that reduced functional connectivity within key networks (e.g., frontoparietal cognitive and attention networks) can limit nociceptive signal integration and alleviate pain. In essence, when regions that evaluate and propagate pain become less synchronized, perceived pain intensity or unpleasantness can decrease (41). Jalon et al. (41) examined cancer patients undergoing neurosurgical procedures for intractable pain and found that both spinal cordotomy (disrupting sensory nociceptive pathways) and cingulotomy (lesioning the anterior cingulate cortex to target affective pain) produced immediate relief and reduced functional connectivity within the salience network (41), supporting the role of salience-network connectivity changes in pain relief. Similarly, VR-based analgesia studies show that patients immersed in VR during painful procedures report large pain reductions and display significantly decreased pain-related brain activity on fMRI (13). These results suggest that VR distraction lessens pain perception by downregulating the connectivity and activity of networks that integrate nociceptive information.

fNIRS-derived biomarkers could identify VR-responsive patients, guide personalized care, reduce pharmacologic burden, and offer a pathway to precise, adaptive, and low-burden pain relief, positioning VR as a safe, scalable, and cost-effective tool in oncology pain management.

## Limitations

This pilot study has some limitations. The single-arm design, without a control or sham VR condition, limits our ability to draw causal conclusions about the specific effects of VR. Although baseline-corrected fNIRS measures were used to reference neural changes to each participant's pre-VR resting state and reduce inter-individual variability, this within-subject approach does not replace the need for a parallel control condition.

The relatively small sample size and the use of a single, short VR session restrict generalizability and do not allow evaluation of long-term analgesic effects. Also, pain intensity was assessed only before and immediately after the VR session, which limits the ability to examine temporal dynamics of pain and brain connectivity changes during the intervention. In addition, although fNIRS enables brain activity to be measured during real-world tasks, it is limited to surface cortical regions and cannot directly measure deeper brain structures such as the anterior cingulate cortex or insula, which play important roles in pain-related networks. Finally, correlation-based analyses identify relationships between variables rather than causal effects, and future studies with larger samples and controlled designs are needed.

## Code availability statement

No custom code or mathematical algorithm was developed for this study.

## Data Availability

The data that support the findings of this study are available from the corresponding author [SBS] upon reasonable request.
